# Investigation of Flammability of Protective Clothing System for Firefighters

**DOI:** 10.3390/ma15072384

**Published:** 2022-03-24

**Authors:** Anica Hursa Šajatović, Sandra Flinčec Grgac, Daniela Zavec

**Affiliations:** 1 Faculty of Textile Technology, University of Zagreb, 10 000 Zagreb, Croatia; 2 TITERA Innovative Technologies, 2212 Šentilj, Slovenia; daniela@titera.tech

**Keywords:** protective clothing for firefighters, flame manikin, fire resistant materials, flammability of materials, thermal decomposition

## Abstract

The main characteristic of clothing for protection against heat and flame is the protection of users from external influences and danger in the conditions of elevated temperatures and exposure to flame, fire, smoke, and water. The paper presents research on the clothing system for protection against heat and flame using a fire manikin and systematically analyses the damage caused after testing. As part of the damage analysis, the existence of microdamage and impurities on the clothing system was determined using a USB Dino-Lite microscope. In addition, the intensities and composition of gaseous decomposition products during the thermogravimetric analysis of samples were investigated. The results of the research using a fire manikin showed that the user of the examined clothing system would not have sustained injuries dangerous to health and life, which confirmed the protective properties. The results of the TG-FTIR indicate that the decomposition of the fabric sample of the modacrylic–cotton fiber mixture takes place in three stages, and the identified gaseous degradation products were H_2_O, CO_2_, and CO.

## 1. Introduction

The most important garment function is to protect the human body from basic outer influences such as wind, rain, sunlight, dust, and mechanical influences. Protective clothing is clothing that protects individuals who are exposed to life threatening or hazardous environments during work [1,2,3]. Today, firefighting is one of the most dangerous occupations because they perform their tasks under specific working conditions that arise in accidental situations, creating and spreading fire. This is why their clothing should protect against extreme conditions such as high temperature and flame, rain and water, cold, mechanical action, aggressive and reactive chemicals, chemicals that are hazardous to health, etc. [4]. Attention should be paid to the usual, most commonly used body positions (standing, squatting or sitting) and extreme movements that are used when wearing clothing and to perform a job [5,6,7,8]. According to the above-mentioned requirements, functional protective clothing against heat and flame should be developed in cooperation with designers, engineers, and firefighters. The process of designing protective clothing includes the entire design activity for the development of new products with high technological content from the initial idea and first project concept to the feasibility analysis, considers new materials and researchers during the design, prototyping, and manufacturing. In addition, in smart and intelligent protective clothing, the integration of technology with textiles creates enormous possibilities. The combination of technology and fashion can be realized only in a multidisciplinary work where engineers, fashion designers, and scientists will work together to adapt to their environment and create a balance between design requirements, function, performance, ergonomics, protection, and comfort [9,10,11].

So far, new types of fire resistance fibers, high performance fiber of special properties, and microporous materials have been developed, which provide for the long life and easy care of protective clothing as well as, at the same time, providing the user with an adequate level of protection and safety [12]. Special procedures of evaluating the characteristics of protective materials, tests with a thermal manikin and hot plates for the determination of fabric and clothing characteristics under special conditions have been developed. Scientists from different fields (textile technology, physiology, ergonomics, functional design, etc.) are continually working on research such as the development and producing of protective clothing and materials, research of their durability during use testing by volunteers in simulated environmental conditions, research on moisture transfer through clothing and thermal stress, and how to design and implement the appropriate test methods [13,14,15,16,17,18].

For clothing in firefighting, fire resistant materials should be used. This could be some fabrics or knits made from aramid fibers such as Nomex^®^, modacrylic fibers, cotton fibers, or other textile materials with a flame retardant finishing. It is often used to make protective clothing against heat, and flame materials can be made of a blend of the aforementioned fibers with the aim of achieving better comfort, which includes the transport of moisture and heat. This is extremely important for achieving comfort when using clothing systems in various activities that include extreme conditions [19]. Recently, different techniques for the characterization of fire resistant textile materials have been conducted [20]. High thermal protection of firefighter clothing systems can be achieved by wearing multilayer or thick textile materials and it is well-known that the performance of each layer of a firefighters’ protective clothing has a significant influence on the level of protection provided. When a clothing system is exposed to high temperature and direct flame or fire, it is still uncertain how destructive different exposures are and how long a piece of firefighting protective clothing can continue to protect to an acceptable level. 

In this paper, a protective clothing system made of carefully selected materials was examined. The underwear is made of a wool/modacrylic blend in a ratio of 70%:30%, which by synergy contributes to high protection and comfort. It is known that the inner core of wool fiber has high moisture-absorbing might and can receive twice the amount of moisture of its mass while remaining dry. This property allows wool to adsorb sweat generated during activity or under the influence of extreme conditions. Precisely because of the internal moisture, wool is naturally resistant to flame, while modacrylic, due to its chemical composition, is characterized by high stability to heat, and the flame has the property of self-extinguishing. The overalls were made of a mixture of modacrylic and cotton in a ratio of 55%:45% in order to achieve high resistance to heat and flame resistance and thermophysical comfort [21,22,23].

Consequently, in this paper, firefighting overalls made from modacrylic/cotton fabric on a fire manikin was tested as well as the mechanical characteristics, and an analysis of damage after fire exposure and the thermal properties of the fabric was conducted.

## 2. Materials and Methods

**Figure 1:**
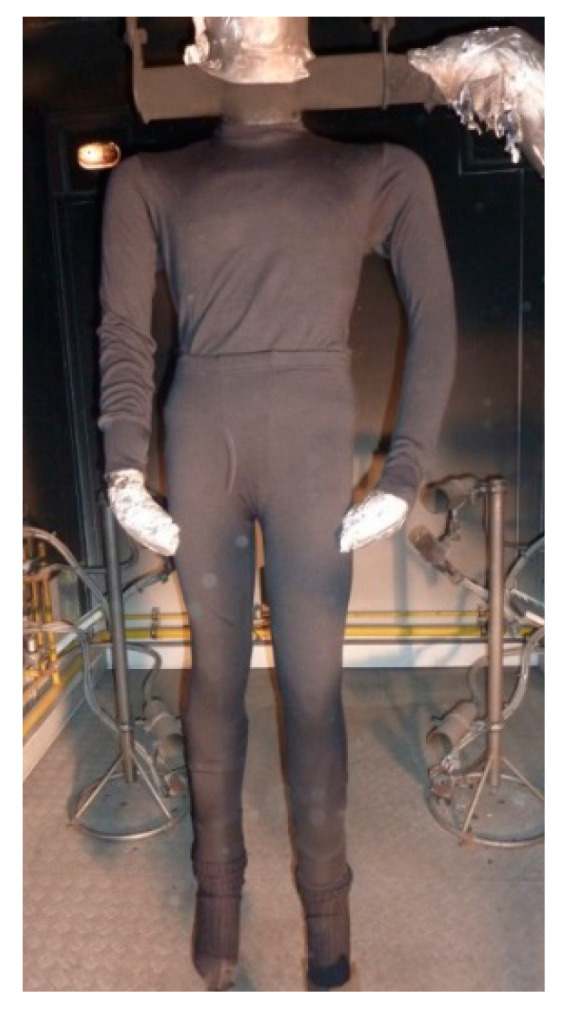
Tested underwear on the fire manikin.

**Figure 2:**
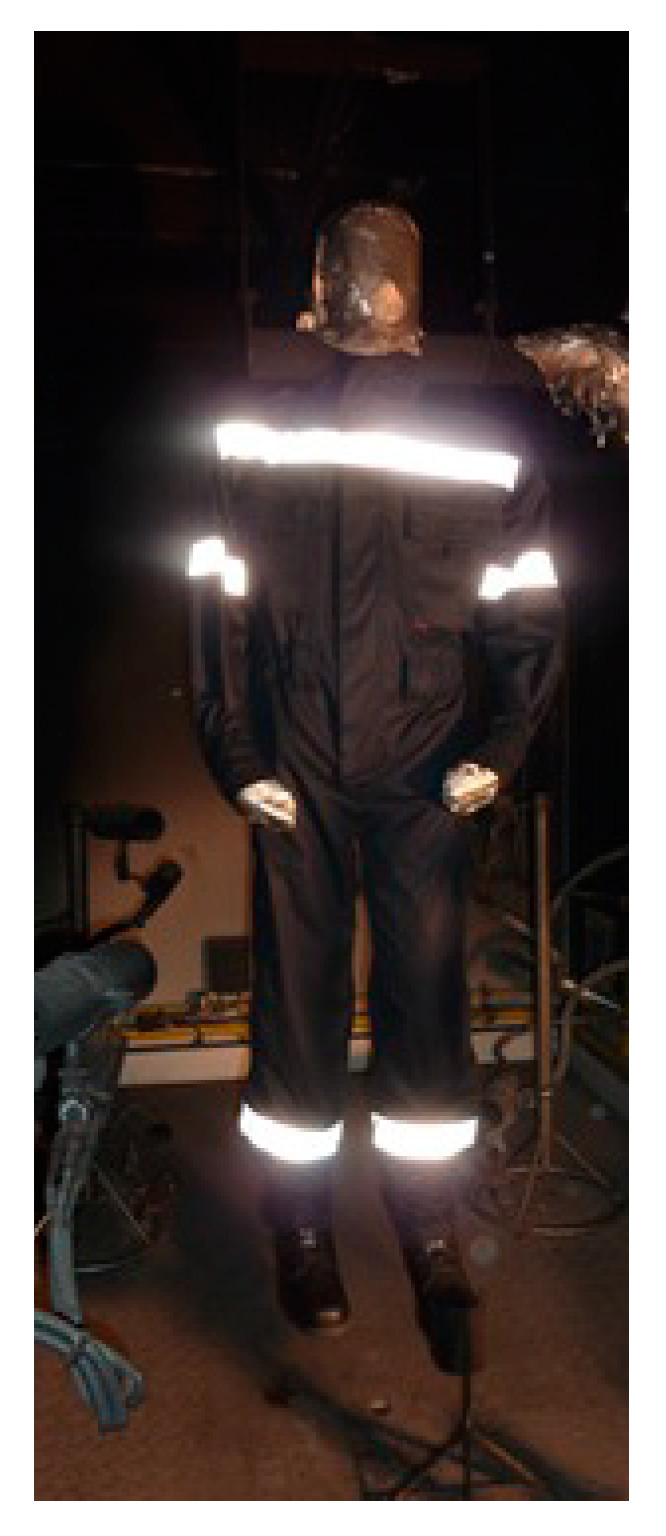
Tested overalls on the fire manikin.

The paper investigated the flammability properties of the clothing system for protection against heat and flame, and analyzed the damage caused to the overalls intended for firefighters to extinguish forest fires after testing by using a fire manikin. A clothing system consisting of underwear composed from 70% wool fiber and 30% modacrylic fiber (Figure 1) and overalls for protection against heat and flame intended for extinguishing forest fires (Figure 2), made of 55% modacrylic fiber and 45% cotton fiber, were tested on the fire manikin.

The following testing instruments and systems were used to conduct the experimental part of the work.

**Figure 3:**
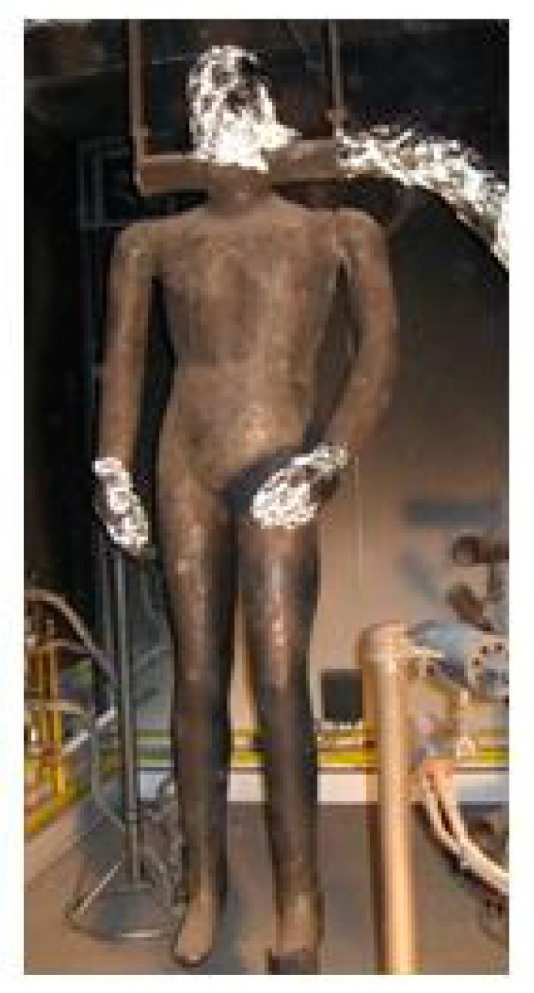
Fire manikin Žiga (Institut Jožef Štefan, Ljubljana, Slovenia).

**Figure 4:**
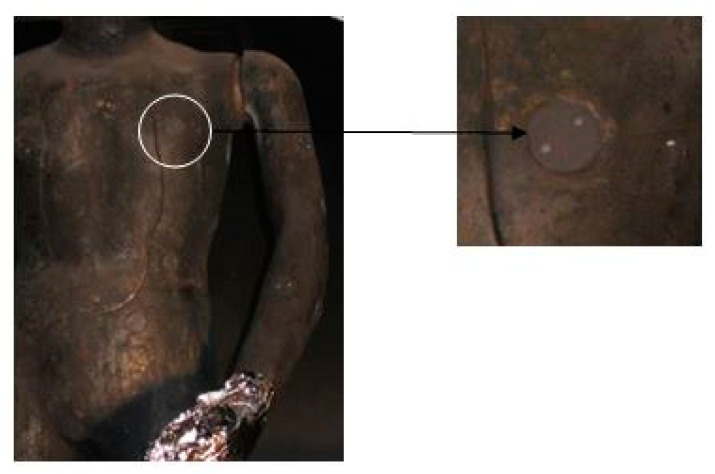
Temperature sensor on the fire manikin.

The fire manikin (Figure 3) was equipped with 128 temperature sensors placed on its surface (Figure 4). The explosive fire simulation system consisted of 12 gas burners located around the fire manikin. Prior to each test, calibration was performed where the naked manikin was exposed to explosive fire for 3 to 4 s. Therefore, burners must be placed appropriately to always provide heat flux values of about 80 kW/m^2^. The data provided by the sensor was collected and displayed using the Labview software solution, and the entire system was controlled by a Mitsubishi Programmable Logic Controller (PLC) unit. Flashfire was achieved by burning the main burners from 2 to 10 s, depending on the duration of the test and the clothing system being tested. Turning off the burner extinguished the fire, and it waits for 120 s until the end of the test when the fan was switched on for faster ventilation of the test room [24].

The clothing system was tested on a fire manikin in accordance with an international standard describing the test method (ISO/DIS13506, 2002). During the test, the clothing system was exposed to open fire for 4 s. Using 128 thermoelements (Figure 4) distributed over the entire surface of the fire manikin (‘skin’), the temperature rises on the ‘skin’ at the time of flame action can be measured. Measurements were recorded every 0.5 s in each area where the thermoelement is located. Based on the temperature data, the heat flux was calculated, which was compared to a human skin model to determine whether burns have occurred. Data were collected for 120 s including the first contact with the flame [20]. After the activity of heat and flame, parts of the overalls were analyzed using a dynamometer, a Dino-Lite USB microscope and a thermal gravimetric device, in order to determine the change in the structure and characteristics of the material. The research was conducted with the aim of determining microdamage and qualitative determination of the present impurities and gases.

**Testing of tensile properties on a dynamometer**

Determination of breaking force and elongation of the fabric in the direction of the warp and weft was carried out by the strip method according to the HR EN ISO 13934-1:2013 standard using a dynamometer Tensolab 3000, tt. Mesdan, Italy. The dynamometer works at a constant stretching speed of 100 mm/min, and the distance between the clamps according to the norm is 200 mm [25].

**Dino-Lite USB microscope**

Technical characteristics of Dino-Lite USB microscope are:

- Magnification: 10–90×, 10–50×/200×, fixed 500×
- Resolution: 1.3 megapixels USB:2.0
- Options: calibration, measurement, photography, video recording
- LED and UV lighting, polarizing filter, diffuse lighting
- Outer materials: composite or aluminum alloy [26].

**Thermal gravimetry (TG) measurements:** A Pyris 1 TGA, PerkinElmer thermogravimetric device measured the loss of sample mass in percent as a function of temperature (and time) during linear or stepwise heating in the temperature range (50 °C–950 °C) and in a certain atmosphere (nitrogen, air, oxygen). The results of TG analysis are presented in the form of curves, and with this method, it is possible to determine the point of degradation or decomposition of the sample. If the device is connected via TG-IR interface (PerkinElmer TL 8000) to an FTIR spectrometer (PerkinElmer, Spectrum 100, Waltham, MA, USA), it is possible to analyze gaseous organic products generated by heating the sample. The sample was heated in a vessel so that the temperature rose evenly at the set rate, while the change in mass was recorded on the balance. Based on the change in mass, it is possible to determine the percentage ratios of the components [25].

Thermogravimetric (TG) experiments were carried out using a Perkin Elmer Pyris 1 TGA thermogravimetric analyzer. Samples were stacked in an open platinum sample pan and the experiment was conducted in an air atmosphere. All samples for TGA were measured from 30 °C to 800 °C at the heating rate of 30 °C/min with a continuous airflow at a rate of 20 mL/min. Samples were studied by the coupled TG-IR technique to better understand the decomposition process of different FR-fabrics. Nitrogen, which does not exhibit IR-absorption, was used as the purge gas, thus the end-products of the decomposition were pyrolyzed rather than the oxidative degraded products. A Thermal Analysis Gas Station (TAGS) equipped with a detector was used for the FTIR analysis. The transfer line, high-temperature flow cell, and TG interface were held at 280 °C for the duration of the run to prevent gas condensation. The evolved gases were transferred through the FTIR flow cell by a peristaltic pump with a flow rate of 60 milliliters per minute. 

**Microscale Combustion Calorimetry Measurement**

The microscale combustion calorimetry (MCC) measurement was performed with a Govmark MCC-2, according to ASTM D7309-2007 (Method A). The fabric samples were first ground in a high-energy vibrating mill RETSCH^®^-MM 400 at a frequency of 25 Hz for 10 min to form homogeneous powders. Sample weight was in the range of 5 to 6 mg. The sample’s thermal degradation products in the nitrogen gas stream were mixed with a 20 cm^3^/min stream of oxygen prior to entering a 900 °C combustion furnace. Each sample was run in three replications and the data presented here are all the averages of the three measurements. The MCC provides the peak heat release rate (PHRR) and heat release capacity (HRC) data of the polymeric sample based on the oxygen consumption, the heating rate of the sample, flow rate, and sample weight [27]. This is extremely important for the characterization of materials intended for the manufacture of protective fire uniforms.

## 3. Results

**Figure 5:**
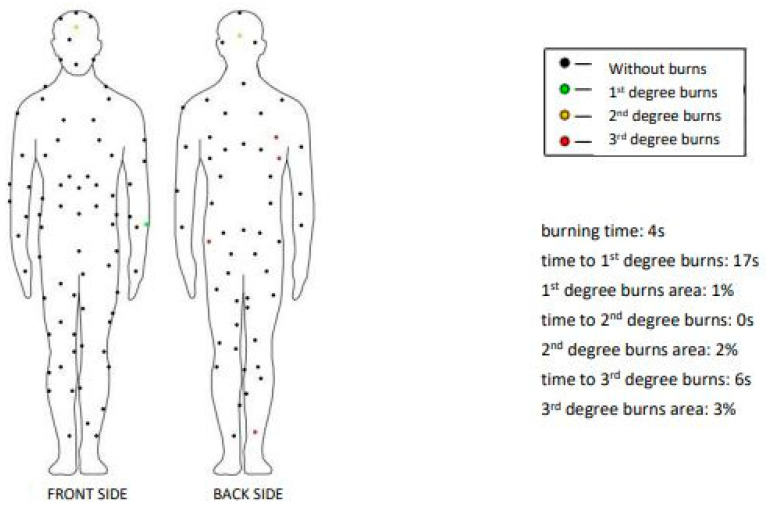
Computer display of the results obtained by the fire manikin test.

By testing with a fire manikin and an explosive fire simulator, data on the degree of burns in the case of using a clothing system (underwear and overalls) that was exposed to an explosive flame for a period of 4 s were obtained (Figure 5).

**Figure 6:**
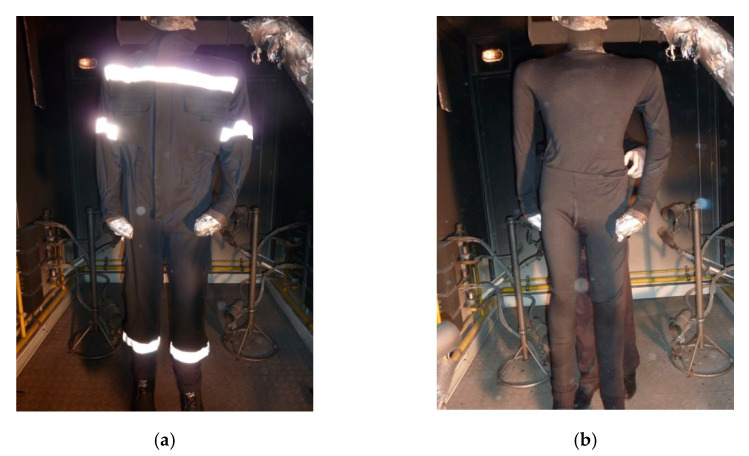
Parts of the clothing system after exposure to heat and flame: (**a**) overalls and (**b**) underwear.

The clothing system consisting of firefighting underwear and a single-layer overalls intended for firefighters to extinguish open fires after being exposed to explosive fire for 4 s was found to have minor damage (Figure 6). Visual assessment of the damage to the clothing system was performed after a period of 116 s after the flame was extinguished in the chamber in which the fire manikin test was performed. Based on the observations, it was established that there was no damage to the underwear (Figure 6b). There was minor damage to the overalls on the tops of the pockets, and on the sleeves and part of the trousers below the knee (Figure 6a). The visual assessment revealed that there was a shrinkage of material on certain parts of the overalls (shoulder area), and a partial change in color on the material of the overalls (dark brown and black) caused by fire (visible on the folds on the overalls, which appeared during the test, Figure 6).

Damage to the clothing system confirmed the results obtained by the computer display of test results on the fire manikin and microscopic analyses, which did not show damage to the structure of the material. The results of the fire manikin (Figure 5) showed that after 17 s, there was the first appearance of first-degree burns in the amount of 1% of the total area of human skin in the left forearm. Second-degree burns occurred on the head but were not considered because the test was performed without head protection (without the use of a helmet and/or undercap). Four sensors indicating third-degree burns were identified as invalid because during the calibration of the fire manikin itself, before the test, it was found that the sensors were out of order. Results from fire manikin and damage to the clothing system showed that the user, who would be exposed to fire and heat, would not have injuries dangerous to health and life, and thus shows that the clothing system met the expected properties of use/protection. If the user used such a clothing system when extinguishing a fire and was exposed to direct fire for 4 s, they would survive without major health problems, but should immediately move away from further heat and flame exposure, which is very difficult in real conditions for extinguishing fires indoors. Since this clothing system is intended to extinguish forest fires and low vegetation fires in nature, it was assumed that the firefighter will not come into direct contact with the indoor fire for more than 4 s, and it can be concluded that the tested clothing system provides sufficient protection. When extinguishing forest fires, firefighters may be exposed to fire and flames, losing the protective properties of the outer layer of the clothing system (overalls), so the user should be careful and must understand the basic properties of clothing worn to protect and prevent possible injuries because extinguishing forest fires can take more than 24 h. According to the presented results, the testing method of the fire manikin is suitable for investigated clothing system.

For the purposes of the research, an analysis of the basic material from which the overalls were made and an analysis of the material after exposure to flame during the test on the fire manikin were made. The basic material was made from 55% modacrylic fiber, 45% cotton fiber, with a built-in antistatic grid, mass per unit area 295 g/m^2^, woven in canvas. The breaking force in the warp direction was 1049 N and 808 N in the weft direction. After exposing the overalls to explosive fire during the test on the fire manikin and visual assessment of the damage, sampling of parts of the overalls in the direction of the warp and weft was performed. The obtained results are shown in Table 1.

In accordance with the visual assessment of the damage to the overalls after exposure to explosive fire, the results shown in Table 1 confirm that the greatest damage and weakening occurred in the area of the trousers of the overalls. The above research proves that if firefighters wear the tested overalls during firefighting and are exposed to direct flames, they should wear long-legged underwear to avoid skin damage and burns. After testing the whole clothing system using a fire manikin, damage was seen only on the overalls, and no damage was seen on the underwear. Due to the above, all further research was conducted only for the firefighting overalls.

**Figure 7:**
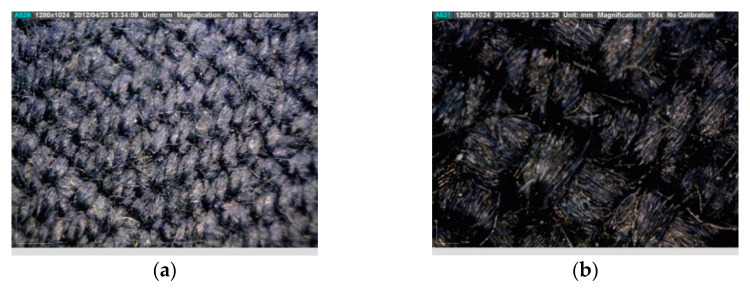
Undamaged sample: (**a**) magnification ×60 and (**b**) magnification ×184.

Analysis of the existence of microdamage and the determination of impurities was conducted with a Dino-Lite digital USB microscope. Figure 7 shows the appearance of an undamaged sample of material from which the overalls were made in two different magnifications with a Dino-Lite microscope (magnification ×60, Figure 7a, and magnification ×184, Figure 7b). An analysis of individual parts of the overalls that were tested on a fire manikin are presented below.

**Figure 8:**
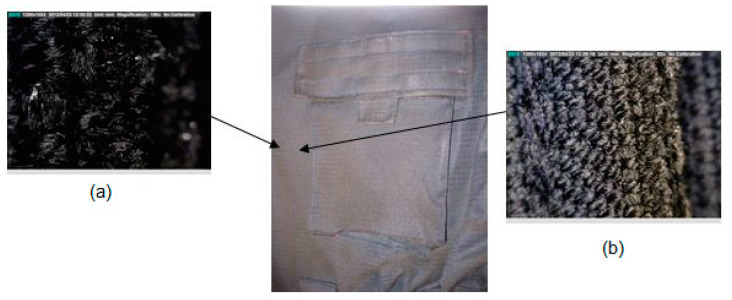
Damage in the area of the upper pocket of the overalls made with a Dino-Lite microscope in two different magnification: (**a**) 184×; (**b**) 60×.

On the cover of the right pocket where the damage was visible, microscopic analysis showed that the material was slightly charred, but there was no damage to the fabric structure itself (Figure 8). It could also be seen that the pocket was functionally shaped and that it expanded and opened only on the lower right side. The pocket cover was also functionally shaped because it was 20 mm larger than the pocket. When making the pocket, non-combustible Velcro tape was used (Figure 8) because there was no damage to the tape or shrinking of the pocket cover after exposure to fire.

**Figure 9:**
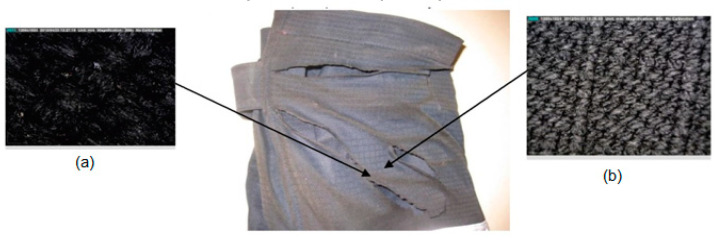
Damage to the sleeve of the overalls made with a Dino-Lite microscope in two different magnification: (**a**) 184×; (**b**) 60×.

On the sleeve, it could be seen that the material was damaged, but microscopic analysis showed that there was no change in the structure of the fabric (i.e., it did not melt, but the fibers under the influence of high temperature were charred and stiffened). Because of this, the material cracked after cooling when removing the suit from the fire manikin (Figure 9). Therefore, after the action of an explosive fire with such damage to the overalls, the user must not continue to extinguish the fire, because in the case of further contact with heat or fire, the overalls will not provide adequate protection.

**Figure 10:**
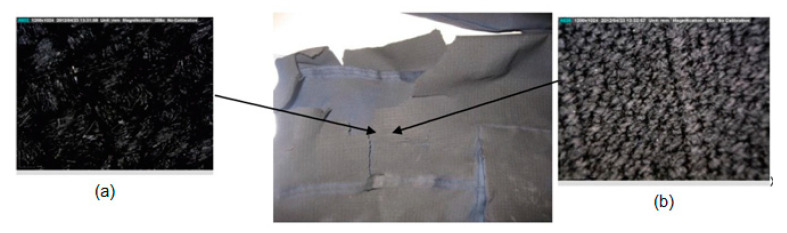
Damage to the left leg of the overalls (lower leg area) made with a Dino-Lite microscope in two different magnification: (**a**) 184×; (**b**) 60×.

Damage to the left leg of the overalls (lower leg area) also occurred during the removal of the overalls from the manikin due to the solidification and charring of the fibers during the exposure of heat and flame (Figure 10). Due to the solidification of the material and its cracking, during further movement of the user, the material would fall off, and such overalls would not provide adequate protection in further use.

**TG analysis with monitoring of gaseous decomposition products of the sample**

Thermogravimetric analysis (TGA) is the most commonly used method to assess the thermal stability of materials. Using a Pyris and TGA thermogravimetric device, (PerkinElmer, USA), the change (loss) of the sample mass as a function of temperature was monitored. Testing using thermogravimetric analysis shows what happens to the material during contact with heat, and what the decomposition products are. 

**Figure 11:**
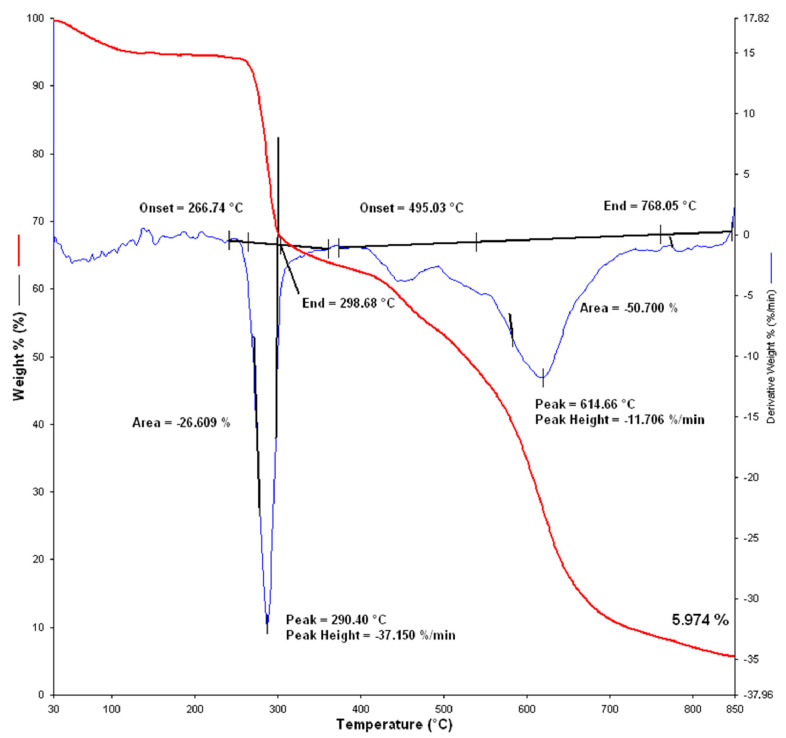
Thermal gravimetric analysis of a sample of the basic material from which the overalls were made (Sample 1) (TG and dTG curve).

**Figure 12:**
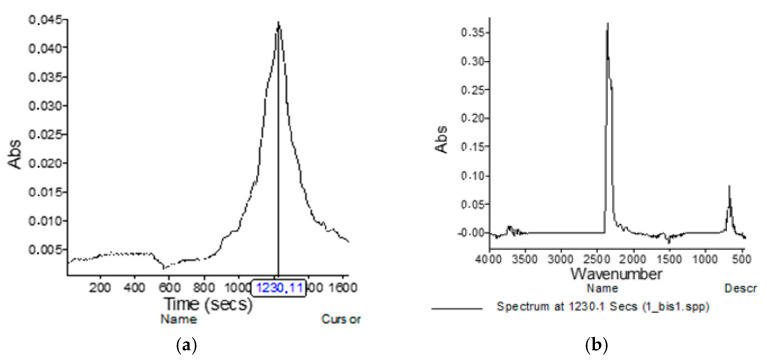
TG-IR analysis of a sample of the basic material from which the overalls were made: (**a**) absorption spectrum of the highest concentration of gaseous decomposition products; (**b**) measured gases on IR at temperature 615 °C.

Figure 11 shows the TG and dTG curves of the thermal decomposition of the basic material from which the overalls was made. From the dTG curve, it can be seen that the degradation of the sample took place in two stages. At a temperature of 290 °C, the first stage of dynamic decomposition was recorded, with a loss of sample weight of 37.150% per minute, and no large quantities of gaseous products were recorded during the decomposition. The second stage of dynamic decomposition began at a temperature of 495 °C, and the peak of the second stage of dynamic decomposition was visible at the temperature of 614 °C (Figure 11), at which a larger amount of gaseous CO_2_ and CO products was detected (Figure 12). The thermal stability of the sample was clearly visible in the residue after thermogravimetric analysis of 5.974% at 850 °C.

**Figure 13:**
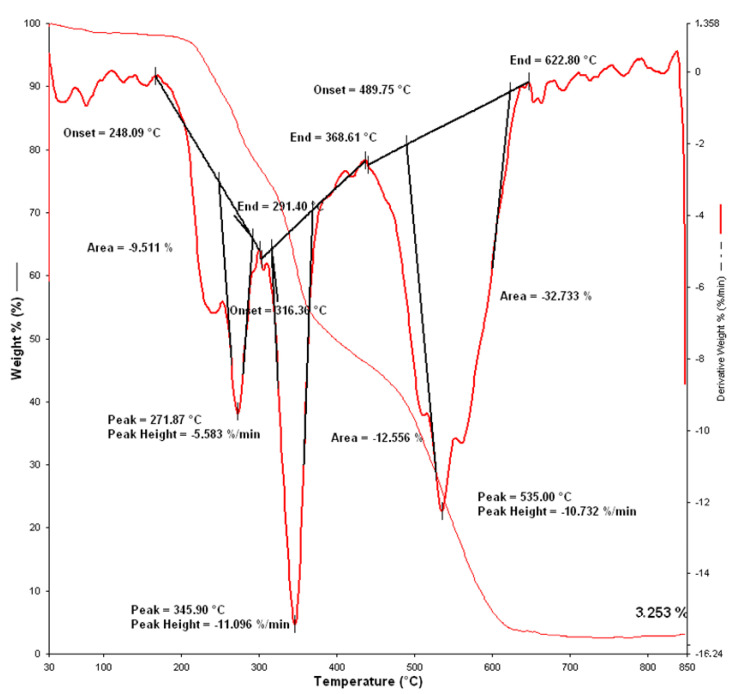
Thermal gravimetric analysis of the material sample of the overalls after exposure to an explosive fire on a fire manikin (Sample 2): TG curve, dTG curve.

According to the obtained TGA curve (Figure 13), it can be seen that the rapid thermal decomposition started at a temperature of 248.09 °C. The thermogravimetric curve of the modacrylic/cotton fiber sample indicates that during non-isothermal decomposition, the sample decomposes in three stages. This was evident from the dynamic degradation of the sample showing the dTG curve. The first stage started at a temperature of 248 °C. If a person without protection is in contact with such a high temperature, they would obtain fourth degree burns, which would lead to irreversible destruction of subcutaneous tissue. According to the results of testing on the Žiga fire manikin, it is evident that a clothing system consisting of fire-resistant underwear and one-layer overalls provides protection, and that the user would suffer first-degree burns, which manifest as redness and mild swelling, without any other skin damage. The maximum rate of dynamic degradation in the first stage was recorded at a temperature of 271.87 °C with a mass loss of 5.583% per minute. Completion of the first stage of decomposition was at a temperature of 291.40 °C. The second stage of dynamic degradation began at a temperature of 316.36 °C, and the maximum dynamic degradation was recorded at a temperature of 345.90 °C. In the second stage of decomposition, the rate of mass loss increased to 11.096%/min. The third stage of dynamic decomposition began at a temperature of 489.75 °C, and at a temperature of 535.00 °C, the maximum dynamic decomposition was recorded with a weight loss of 10.732%/min. The residue after thermogravimetric analysis for the specified sample was 3.253%, which means that the sample in the specified temperature range lost 96.7% of its mass.

**Figure 14:**
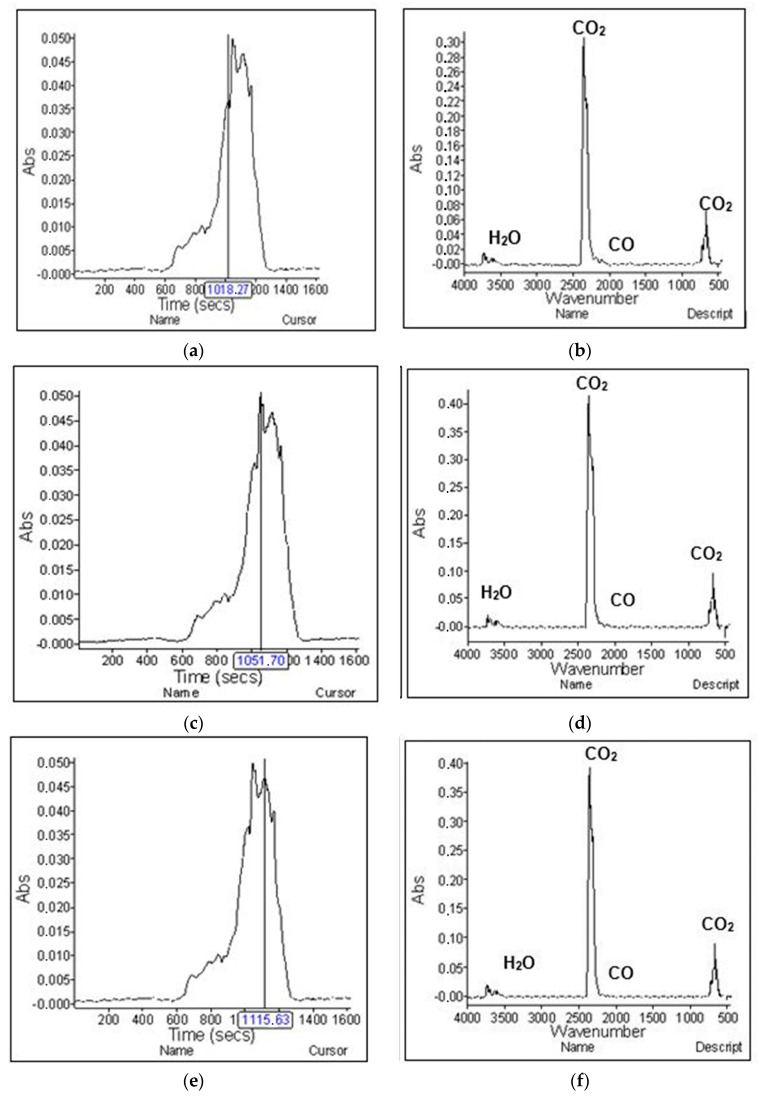
TG-IR analysis of sample. (**a**) The first intense absorption spectrum; (**b**) measured gases on IR at a temperature of 509.13 °C: (**c**) second absorption spectrum; (**d**) measured gases on IR at a temperature of 525.85 °C; (**e**) third absorption spectrum; (**f**) measured gases on IR at a temperature of 557.82 °C.

Figure 14 shows the gaseous degradation products of the modacrylic/cotton fiber sample (Sample 2). The first intense absorption peak was detected at 509.13 °C (Figure 14d). Evaporative decomposition products were identified as CO_2_ (characteristic highest points at 2359 and 2322 cm^−1^), CO (characteristic highest points at 2179 and 2110 cm^−1^), and water (characteristic highest points at 3500 to 4000 and 1550 to 1566 cm^−1^). The second intense absorption peak was measured at 525.85 °C, and the third intense absorption peak was measured at 557.82 °C. The same decomposition gases were detected in all of them.

In order to gain a better insight into the behavior of the sample under the action of heat and flame, research was conducted on a microscale combustion calorimeter (MCC). The obtained results (Table 2) indicate that Sample 1 was thermally more stable than Sample 2. It can be seen that the recorded specific heat release capacity, which for Sample 1 was 34.67 J(g∙K)^−1^ and Sample 2 was 45.33 J(g∙K)^−1^, and the maximum specific heat release was 26.13 W∙g^−1^ in Sample 1 and 37.68 W∙g^−1^ in Sample 2. The lower the value of the maximum specific heat release (Qmax), the more stable the sample is to the action of heat and flame. The sample previously damaged by heat and flame during the test on the fire manikin showed slightly less stability compared to the same material before the test. It is assumed that the reason for this is the weakened modacrylic component, which has the role of preventing the development of heat and complete combustion of the cellulosic component. Re-exposure of previously thermally weakened material (Sample 2) to heat and flame resulted in complete combustion of the cellulosic component of the material and partially modacrylic component, which was clearly visible through the amount of charred residue relative to the undamaged one-layer overalls (Sample 1) [19,28].

## 4. Conclusions

Based on theory and conducted research, except for the important factor that is the material selection, great attention should be paid to the functional design of clothing for protection against heat and flame. The tested clothing system provides a high degree of protection, which was in part due to the adequate use of fire-resistant underwear. The results of testing using the fire manikin showed that when using such a clothing system, the user would not suffer tissue damage, which could pose a danger to health or life, if the users are immediately removed from the fire and heat. If the user continues to use a clothing system that has been exposed to explosive fire, the clothing system would not provide adequate protection due to the damage. Without the use of fire-resistant underwear, the user would obtain burns in more areas than with the fire-resistant underwear, especially if they used ordinary cotton underwear, which absorbs moisture faster and the user would “be cooked“ when exposed to high temperatures. If the user was exposed to prolonged fire, the damage to the overalls and the injuries sustained would be greater. In practice, when extinguishing fires in forests and low vegetation, such interventions last more than two days under high temperatures, so the studied clothing system would not provide adequate protection if users are exposed to explosive fire.

Research with the help of a fire manikin and an explosive fire simulator (i.e., simulations of the dangers to which firefighters are exposed), greatly helps us to predict the degree of burns of users of the clothing systems, and the possibility of survival. Such research is already of great importance in the first phase of the design of clothing systems, which should provide protection against heat and flame. It is very important that all of the used materials are fireproof, so that the failure does not further endanger the health or life of users. In the design and production processes of protective clothing systems, the cooperation of experts in the field of materials and fibers, designers, constructors, and technologists as well as the end users and manufacturers of protective clothing is necessary. 

From the obtained TG-IR and MCC results, it can be concluded that the thermal properties of the protective firefighting clothing system decreased after the exposure to thermal manikin testing (Sample 2) compared to the starting material (Sample 1). However, both tested materials showed relatively good stability against the heat, but based on all of the measured indicators, it requires further development to improve the overall thermal resistance properties.

## Figures and Tables

**Table 1:** Results of the tensile properties of basic materials (before heat exposure) and of the part samples of the overalls after exposure to explosive fire on a fire manikin.

	Direction	Breaking Force [N]	Breaking Elongation [%]	Breaking Time [s]
Basic material	Warp	1049	22.65	27.2
	Weft	808	15.96	19.2
Samples of overalls material after exposure to explosive fire on a fire manikin				
Underside of trousers	Warp	180	13.742	16.5
	Weft	94	5.890	7.1
Back part of overalls	Warp	681	34.366	41.5
	Weft	383	19.244	23.2
Topside of trousers	Warp	182	9.960	12.0
	Weft	92	5.210	6.5

**Table 2:** Results of the measurement parameters of the samples of parts of the overalls on a microcalorimeter for combustion.

Measurement Parameters	Sample 1 (Before Exposure to Fire)			Sample 2 (After Exposure to Fire)		
	$\bar{x}$	s	CV, %	$\bar{x}$	s	CV, %
Heat release capacity, ηc J∙g^−1^∙K^−1^	34.67	0.577	0.016654	45.33	1.528	0.033695
Maximum specific heat release, Qmax, W∙g^−1^	26.13	0.220	0.008404	37.68	4.894	0.129864
Heat release temperature, Tmax, °C	386.3	2.326	0.006021	440.03	3.550	0.008067
Specific heat release, hc, kJ∙g^−1^	6.2	0.1	0.016129	6.23	0.153	0.024505
Yield of pyrolysis residue, Yp, g∙g^−1^	0.45	0.012	0.026012	0.33	0.008	0.025397
Specific heat of combustion of fuel gases, hc, gas, kJ∙g^−1^	11.34	0.295	0.026009	9.35	0.144	0.015381
Average release capacity, ηc, J∙g^−1^∙K^−1^	41.33	0.577	0.013968	51.67	1.528	0.029565
Average release temperature, Tmax, K	296.3	5.246	0.017705	331.33	1.401	0.004228

## Data Availability

Not applicable.
